# A scoping review of gaze and eye tracking-based control methods for assistive robotic arms

**DOI:** 10.3389/frobt.2024.1326670

**Published:** 2024-02-19

**Authors:** Anke Fischer-Janzen, Thomas M. Wendt, Kristof Van Laerhoven

**Affiliations:** ^1^ Faculty Economy, Work-Life Robotics Institute, University of Applied Sciences Offenburg, Offenburg, Germany; ^2^ Ubiquitous Computing, Department of Electrical Engineering and Computer Science, University of Siegen, Siegen, Germany

**Keywords:** robot, assistive robotics, input modalities, eye tracking, assisted living, EOG, hybrid BCI, human robot interaction (HRI)

## Abstract

**Background:** Assistive Robotic Arms are designed to assist physically disabled people with daily activities. Existing joysticks and head controls are not applicable for severely disabled people such as people with Locked-in Syndrome. Therefore, eye tracking control is part of ongoing research. The related literature spans many disciplines, creating a heterogeneous field that makes it difficult to gain an overview.

**Objectives:** This work focuses on ARAs that are controlled by gaze and eye movements. By answering the research questions, this paper provides details on the design of the systems, a comparison of input modalities, methods for measuring the performance of these controls, and an outlook on research areas that gained interest in recent years.

**Methods:** This review was conducted as outlined in the PRISMA 2020 Statement. After identifying a wide range of approaches in use the authors decided to use the PRISMA-ScR extension for a scoping review to present the results. The identification process was carried out by screening three databases. After the screening process, a snowball search was conducted.

**Results:** 39 articles and 6 reviews were included in this article. Characteristics related to the system and study design were extracted and presented divided into three groups based on the use of eye tracking.

**Conclusion:** This paper aims to provide an overview for researchers new to the field by offering insight into eye tracking based robot controllers. We have identified open questions that need to be answered in order to provide people with severe motor function loss with systems that are highly useable and accessible.

## 1 Introduction

### 1.1 Motivation and research questions

Assistive robotics is a broad field that describes the use of robots to assist the elderly or people with physical and cognitive disabilities. The field describes several types of robotic applications. For example, social, service and surgical robots, walking aids, exoskeletons, power wheelchairs, therapy robots and assistive robotic arms. The term Assistive Robotic Arm (ARA) is only one of several keywords used in the literature. The rationale for this work is based on the versatile naming found in various publications. Robotic arms used to assist people in everyday life have been called, among others, wheelchair-mounted robotic arm, assistive robotic manipulator and assistive robot, which complicates the retrieval of related work. In this paper, we will use the term Assistive Robotic Arm.

Controlling a robot with eye movements could be an appropriate solution for people with severe physical impairments of arms, head movement, and speech. In Germany, for example, 7.8 million people are severely disabled. 11% have disabilities of the arms and legs, 10% of the spine and upper body, and 9% have a cerebral disorder (Destatis, 2022). This results in approximately 2.3 million people who could benefit from an eye tracking control. Locked-in patients in particular could benefit from an ARA. Locked-in Syndrome can result from brainstem lesions such as stroke, traumatic brain injury, and tumors, from brainstem infections or degenerative diseases. Depending on the individual case rehabilitation therapy can restore body functions (Smith and Delargy, 2005). Amyotrophic Lateral Sclerosis (ALS) is a progressive disease that immobilizes the patient over time. In the Locked-In state and in the late stages of ALS, there is a high probability that the motor function of the eye is still intact, showing possibilities for the use of eye tracking (Edughele et al., 2022).

Currently, robots are used primarily in industrial applications to automate work. Adapting such robots as assistive systems requires interdisciplinary knowledge due to the variety of daily tasks and environments. Such tasks can be grouped into Activities of Daily Living (ADL) (WHO, 2001). They involve routines such as cleaning, eating, and personal hygiene, to ensure a good quality of life. Today, ARA can be controlled by joysticks. In these cases, the 3-dimensional moving robot is controlled solely by the user. Shared control, where Artificial Intelligence (AI) is used to reduce the cognitive load of the user in complex tasks, is being explored (Bien et al., 2003; Aronson et al., 2021). However, concerns about Human Robot Interaction (HRI) and safety are raised due to the close proximity to the user (Bien et al., 2003). A concern in assistive robotics is to ensure the wellbeing of the user. Among the challenges of realizing the various applications and ensuring the safety of the user, the usability and accessibility of ARAs must be addressed. If the system is not easily accessible and adapted to individual needs, people tend not to use it. A related challenge is the Midas touch problem, which describes the misinterpretation of gaze that triggers robot commands. This leads to user frustration (Alsharif et al., 2016; Di Maio et al., 2021). This challenge is compounded by the need to control the robot in a 3-dimensional space using 2-dimensional eye movements. For these reasons, this work will address the research questions in Table 1.

**TABLE 1:** Research questions and motivation.

No.	Research question
RQ1	What approaches have been explored in the field of gaze-controlled robotic arms to assist people with (severe) upper limb impairments?
RQ2	How can eye gaze be interpreted to control a robot?
RQ3	What are the developments, limitations and challenges that can be found in the field of gaze-controlled assistive robotic arms?

The main contributions of this work are based on the answers to these questions.

1. An overview of publications in the heterogeneous field of eye movement based robot control.
2. A detailed specification of the technology used (Anke Fischer-Janzen, Study Descriptions, URL: https://github.com/AnkeLinus/EyeTrackingInRobotControlTasks/blob/main/StudyDesciption.md, last accessed: 09.02.2024) and the studies conducted (Anke Fischer-Janzen, Overview of Measurements, URL: https://github.com/AnkeLinus/EyeTrackingInRobotControlTasks/blob/main/OverviewOfMeasurements.md, last accessed: 09.02.2024)
3. Future trends and open questions in eye-movement based robot control and a comparison of approaches

### 1.2 Technical background

Eye movements are used in a variety of assistive technology applications. Popular are eye typing interfaces that provide the user with the ability to speak and eye mouse that allows the person to use a computer (Al-Rahayfeh and Faezipour, 2013). Other applications include the control of electric wheelchairs that provide mobility to the user (Cowan et al., 2012). Social robots use it to extract facial features and interpret the feelings and needs of the user (Kyrarini et al., 2021).

Fixation and saccades are the most common eye movements. Fixation describes the gaze resting on a particular point. The duration of fixation depends on the object or location being fixated and ranges from tens of milliseconds or seconds. Fixations can be measured as dwell time, which is the amount of time a user fixates on an object. This is often used as a parameter to interpret the user’s intent or to give a command to the robot. Saccades are rapid movements of the eye. They last only a few milliseconds and can be as fast as 500°/*s* (Holmqvist and Andersson, 2017). These numbers vary in the literature literature. This may be due to the fact that everyone is anatomically and behaviorally unique, including how they move the eyes.

As the environment and the person move or the depth of field changes, the eye responds with vergence movements and smooth pursuit. Vergence movements describe the movement of the eye during reading. The axis of the eyes moves from parallel (distance vision) to an intersection on the page (near vision). This prevents double vision. Smooth pursuit describes the eye movement while looking at an object and moving the head or the object itself. This eye movement is not voluntarily. The slow speed of the eye with less than 30°/*s* in smooth pursuit distinguishes it from saccades (Holmqvist and Andersson, 2017). Most of the systems evaluated use fixation on objects or directions, but in head-mounted eye tracking devices smooth pursuit could have an impact on the function of the system.

There are several techniques to measure these movements. The techniques can be divided into Infrared Oculography (IOG), Video Oculography (VOG), Electrooculography (EOG), and electromagnetic coils (Klaib et al., 2021). All techniques are used to measure vertical and horizontal eye movements. Depending on the system, additional movements such as eyebrow movements, blinks, and pupil dilation can be recorded.

**FIGURE 1:**
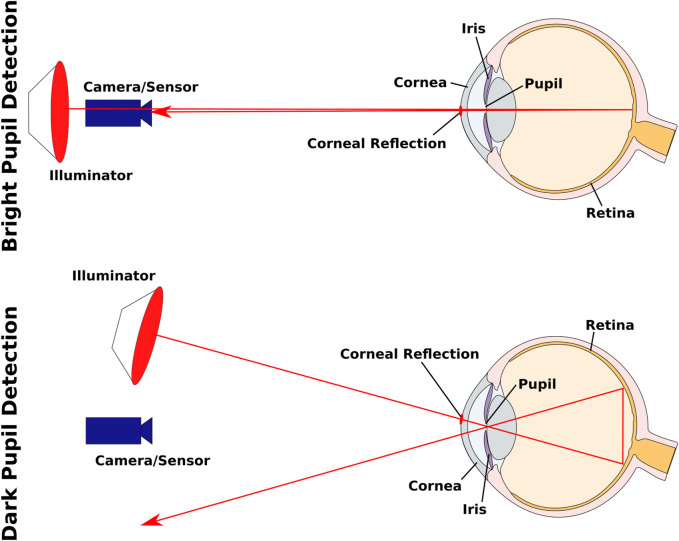
A comparison between bright pupil and dark pupil detection. The corneal reflection used for determining the Pupil Corneal Reflection Point is shown on the cornea. Adapted from Tobii (2023), the anatomy is presented in a simplified form.

IOG uses infrared sensors to detect IR light. Unlike VOG, which records visible light, the invisible light is less distracting to the user, especially in darker environments. Most eye trackers require additional illuminators to create the following effect. When using Pupil Corneal Reflection Point effect, illumination creates a reflection on the cornea. By tracking the vector between the center of the pupil and this reflection eye movement can be determined (Klaib et al., 2021). As it will be presented, most eye trackers are based on infrared light because it provides more contrast of the pupil. This is based on the dark pupil detection method, where by illuminating the eye with IR light, the pupil appears darker than the iris (Edughele et al., 2022). Another way to achieve this effect is to place the illuminator at a different angle to the camera (see Figure 1). With this method, the light reflected from the retina is blocked by the iris, resulting in a darker pupil. In contrast, for bright pupil detection the illuminator is placed close to the optical axis of the camera, creating what is known in photography as the red-eye effect. The light reflected from the retina is captured by the camera (Tobii, 2023). Depending on the algorithm, the appearance of the pupil contrast can lead to better measurement results. With IOG and VOG it is possible to measure the pupil size in addition to the horizontal and vertical movements of the eye, as is done in behavioral studies (Tobii, 2023).

EOG measures corneo-retinal standing potentials by detecting the electric field and is used to measure horizontal, vertical eye movements and eyebrow movements. This method is based on placing multiple electrodes on the user’s face in specific regions. Changes in the electric field are generated by the eye movements (Klaib et al., 2021). These systems can be used with a hybrid Brain Computer Interface (hBCI). A Brain Computer Interface (BCI) uses Electroencephalography (EEG) to measure neural signals. EEG is a mostly non-invasive neuroimaging technique used to measure and record the electrical activity generated by the brain through electrodes placed on the scalp. These signals can be used to interpret the user’s intention and to control external devices such as speech computers and robots (Chaudhary et al., 2022; Karas et al., 2023). hBCIs can use additional inputs such as EOG to improve the quality of the measurement, as will be presented in the results. For example, in Huang et al. (2019) Steady-State visually Evoked Potential (SSVEP) is used to control the system. SSVEP is an electrical signal evoked by the brain’s response to a visual flickering stimulus that has a constant frequency. From this pattern, the brain generates generative oscillatory electrical activity that can be measured by EEG.

Since no related publications using electromagnetic coils were found in this work, we refer to Holmqvist and Andersson (2017), Klaib et al. (2021), and Edughele et al. (2022) for further information. These techniques are used to classify the retrieved publications as shown in Table 4. There will be occasions when a system is labeled as VOG/IOG. No detailed constraints could be found in literature for the eye tracker technique used. This is possible with both infrared and visible light.

## 2 Materials and methods

This review was conducted in accordance with the PRISMA 2020 Statement. Due to the heterogeneity of the literature the PRISMA-ScR extension (Tricco et al., 2018) was used to present the results. In terms of the research questions, this review focuses on eye tracking based control for robots. In the following the databases, search terms, inclusion and exclusion criteria are listed below.

### 2.1 Search strategy and selection process

Three databases listed in Table 2 were searched to identify publications of interest. During the planning phase, non-standardized Internet searches were conducted as a preliminary evaluation to gather further information and identify search terms, including commercial systems and patents. After the systematic identification and screening process, a snowball search was conducted to identify relevant publications from other databases such as Scopus. Publications in English and German were included in the review process.

**TABLE 2:** Databases used in the identification phase.

Data base	Available at	Last accessed
IEEE	https://ieeexplore.ieee.org/Xplore/home.jsp	07.07.2022
ACM	https://dl.acm.org/	01.02.2023
MDPI	https://www.mdpi.com/	01.02.2023

**FIGURE 2:**
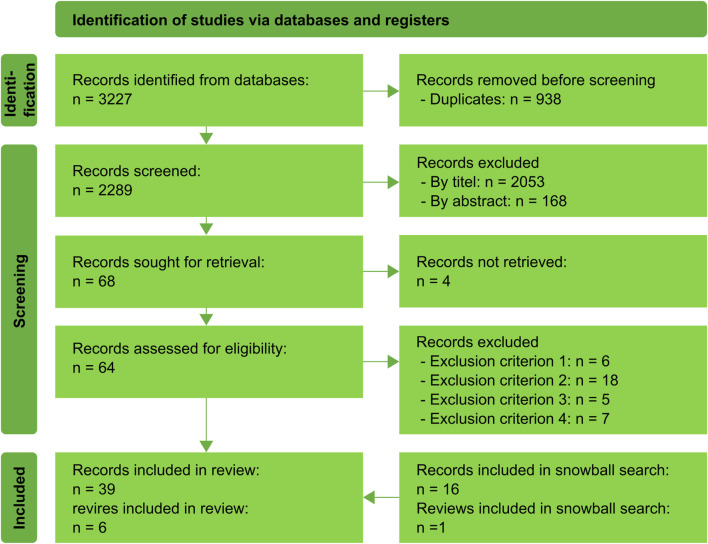
The state flow shows the identification and screening process according to PRISMA 2020.

The search terms used for identification were permutations of the words “eye tracking,” “robot,” “eye,” “shared control,” “gaze” and “assistive.” Combinations of two search terms such as “eye” AND “robot” were neglected due to the number of publications found from other research topics outside the scope. As can be seen in Figure 2, the vast majority of reports were manually excluded due to ambiguous terms. Searching for “assistive robotic arms” (ARA) or “wheelchair mounted arms” (WMRA), which would imply an aid for physically disabled people, would have excluded most of the relevant papers. The general use of these terms is not common and varies between disciplines. Similar results can be found for eye and gaze used as synonyms, leading to ambiguous results for robotics, such as “eye-in-hand” describing the use of a camera mounted on the robot’s end effector to improve automated grasping.

### 2.2 Inclusion and exclusion criteria

Table 3 shows the inclusion and exclusion criteria. Publications were included in the evaluation if the robot was controlled by eye movements. Accordingly, robot type and eye tracking method were included as evaluation items in Table 4. Any system used in a non-assistive task was excluded. This decision led to the inclusion of industrial applications with shared workspaces, as the system could also be used by people with motor impairments in their working life. Other excluded applications can be summarized as surgical assistance systems and telepresence robots in hazardous environments. In these cases the robot is not specifically designed to assist people with physical disabilities. Rehabilitation robots are used to mobilize the person. In such tasks, the robot’s movements are adapted to meet the patient’s anatomy. Visual feedback can be helpful to improve the rehabilitation process. However, as it will be shown the application of eye tracking varies greatly and many systems are exoskeletons. An exoskeleton is not controlled by gaze alone. To ensure safe use, torque, force, and pressure sensors are used to prevent unhealthy forces on the body. Rehabilitation robots are usually limited to a certain movement set, which is different from the wide range needed in everyday life. Therefore, the architecture of a rehabilitation system is different from an ARA, making a valid comparison impossible. The first research question depends on several variables: the space in which the robot can move, the level of automation and whether the systems presented have been tested by users (able-bodied or disabled). If no information was provided, this did not lead to exclusion due to the small number of publications included.

**TABLE 3:** Inclusion and exclusion criteria.

No.	Inclusion criterion
1	A robotic arm is controlled by eye tracking, regardless of the type of robot, the eye tracking method, or the area of use
2	Eye tracking is used as a control input for a robotic arm among other control inputs
No.	Exclusion criterion
1	The type of publication was a bachelor’s, master’s, or doctoral thesis, a patent, or a commercial system
2	When eye tracking was used to control something else than a robotic arm, or the control of the robotic arm was only implied
3	Eye tracking was used as a monitoring device only
4	Publications in which the robot was used as an exoskeleton or as a therapeutic robot

**TABLE 4:** List of articles included. Eye tracking devices were divided into VOG, IOG, and EOG. Robotic arms were distinguished between their use in assisted living (ARA), industrial (R), educational (EDR) or collaborative robots (CR). Eye movements are described as fixations (F), saccades (S), blinks (B) and winks (W) and SSVEP (E). The last three columns describe the number of able-bodied (AP) and disabled participants (DP), and trials (T) in the user study. More information can be found in the tables provided in Fischer-Janzen (2023).

Author, year	Technology			Motion	Eye Movem	Robot Space	AP	DP	T
Telemanipulation									
Kim et al. (2001)	VOG	Head-worn	R	Point	F	3D	—	—	—
Yoo et al. (2002)	IOG	Remote	n.s	Point	F	3D	—	—	—
Bien et al. (2003)	IOG	Head-worn	R	Task	n.s	n.s	—	—	—
Bien et al. (2004)	IOG	Head-worn	R	Task	F	3D	0	6	20
Iáñez et al. (2010)	EOG	Head-worn	R	Point	S	2D	3	0	n.s
McMullen et al. (2014)	IOG/VOG	Remote	PR	Grasp	F	3D	2	0	28/31
Webb et al. (2016)	IOG	Remote	ARA	Grasp	F	3D	5	0	5
Zeng et al. (2017)	IOG	Remote	R	Task	F	3D	8	0	30
Jones et al. (2018)	VOG	Remote	n.s	Task	F	2D	20	0	9
Huang et al. (2019)	EOG	Head-worn	ARA	Task	B + E	3D	5	0	3
Sharma et al. (2020)	IOG	Remote	EDR	Task	F	3D	9/6	9/6	2/2
Stalljann et al. (2020)	IOG	Head-worn	CR	Task	F	3D	10	1	33
Di Maio et al. (2021)	IOG	Remote	R	Point	F	3D	7	0	3
Scalera et al. (2021a)	IOG	Remote	CR	Task	F + S	3D	—	—	—
Scalera et al. (2021b)	IOG	Remote	CR	Task	F + S	3D	—	—	—
Sunny et al. (2021)	IOG	Remote	R	Task	F	3D	10	0	15
Dragomir et al. (2021)	IOG/VOG	Remote	CR	Task	F	3D	—	—	—
Sharma et al. (2022)	VOG	Remote	EDR	Task	F	n.s	7	6	1
Directional gaze									
Bannat et al. (2009)	IOG/VOG	Head-worn	R	Task	F	3D	—	—	—
Ubeda et al. (2011)	EOG	Head-worn	R	Point	F	2D	6	0	n.s
Khan et al. (2012)	EOG	Head-worn	R	Task	B	3D	—	—	—
Zhang et al. (2013)	EOG	Head-worn	R	Point	S	2D	4	0	n.s
Wang et al. (2015)	IOG/VOG	Remote	n.s	Grasp	F + P	n.s	8	0	n.s
Alsharif et al. (2016)	IOG/VOG	Head-worn	CR	Task	F + B	3D	10	1	1
Dziemian et al. (2016)	IOG	Remote	CR	Task	F + W	3D	8	0	5
Wang et al. (2018)	IOG	Remote	EDR	Point	F	2D	8	0	30
Perez Reynoso et al. (2020)	EOG	Head-worn	n.s	Point	F	3D	30	n.s	20
Rusydi et al. (2014a)	EOG	Head-Worn	n.s	Point	F	2D	3	0	20
Rusydi et al. (2014b)	EOG	Head-worn	n.s	Point	F + B	2D	3	0	n.s
Object-oriented gaze									
Onose et al. (2012)	IOG	Head-worn	ARA	Task	F	3D	0	9	var
Huang and Mutlu (2016)	IOG/VOG	Head-worn	ARA	Grasp	n.s	3D	26	0	1
Tostado et al. (2016)	IOG	Remote	EDR	Grasp	F + B	3D	7	0	n.s
Catalán et al. (2017)	IOG	Head-worn	ARA	Grasp	F	3D	—	—	—
Li et al. (2017)	IOG	Head-worn	ARA	Grasp	F	3D	n.s	0	4
Ivorra et al. (2018)	EOG,IOG	Head-worn	ARA	Grasp	F	3D	10	0	20
Cio et al. (2019)	VOG	Remote	ARA	Grasp	F	3D	—	—	—
Wöhle and Gebhard (2021)	IOG	Head-worn	CR	Point	F + B	3D	3	0	10
Yang et al. (2021)	IOG	Head-worn	PR	Point	F	3D	5	0	5
Park et al. (2022)	IOG/VOG	Head-worn	CR	Grasp	F	3D	—	—	—

### 2.3 Data charting process and data items

The data chart presented in Table 4 and online was constructed iteratively. Variables were selected with respect to author information, hardware and software setup, and empirical study criteria. The rationale for the chosen parameters was to find parameters that would allow the reader to gain insight into the design and development of eye tracking based ARA and, if already developing such a system, to find similar approaches. While reading the included publications the importance of the selected parameters for the original authors was estimated and the table was adapted. This resulted in two tables with 17 (Anke Fischer-Janzen, Study Descriptions, URL: https://github.com/AnkeLinus/EyeTrackingInRobotControlTasks/blob/main/StudyDesciption.md, last accessed: 09.02.2024) and 12 (Anke Fischer-Janzen, Overview of Measurements, URL: https://github.com/AnkeLinus/EyeTrackingInRobotControlTasks/blob/main/OverviewOfMeasurements.md, last accessed: 09.02.2024) elements respectively. Since these tables were too large to fit in this paper, it was decided to extract the most important ones, presented in Table 4, to facilitate the reading of the paper. For the author information four items were selected (names, year, title, and DOI). The system information was divided into six items (technology overview, eye movement detection device, eye tracking technique and sensors, wearable or remote eye tracking device, robot manipulation space dimensions, and type of robot). The software was described in three parameters (algorithms and models used in the approach, programming environment, type of eye movement used to control the robot). Finally, the empirical parameters were specified in four parameters (task description, number of participants, number of repetitions, empirical test performed) and additional three parameters describing the specified measurements and parameters divided into task related, computational and empirical parameters. This list was prepared by the first author and discussed with all authors.

### 2.4 Critical appraisal and synthesis methods

The interpretation of the results is limited to the statements of the cited literature. The list may not be exhaustive for research areas beyond eye tracking techniques in the context of robotics based on the search terms. As indicated by several authors, telemanipulation of robots includes various control input devices. Related research on input modalities such as joysticks may provide different perspectives to the discussion presented here. A risk of bias may exist for some of the evaluation items. Multimodal control and shared control exist for other areas of research. For example, gaze-based control is increasingly used to control electric wheelchairs and computer interfaces. An in-depth look at algorithmic design proposals is beyond the scope of this paper and not feasible due to the heterogeneity of the included work. However, the question arises to what extent the results are transferable. In order to reduce the risk of bias, reviews on the aforementioned topics were reviewed, interpreted, and included in the discussion.

The retrieved data will be evaluated in terms of challenges, benefits, limitations, and identified research interests. The challenges, benefits, and limitations are extracted and generalized in the case of duplicates. Sources will be provided for each summarized item. The research interests are extracted by sorting the publications according to their aim and contribution. Three timelines were created for three categories: telemanipulation, directional gaze, object-oriented gaze. Keywords were evaluated to indicate category membership.

The presentation of articles varied greatly depending on whether the researcher was working in computer science, robotics, or behavioral science. Therefore, research interests were extracted through a summary of keywords resulting from these parameters to reduce the influence of personal and field bias. Author defined keywords were collected and ranked according to their frequency of occurrence.

## 3 Results

The adapted PRISMA 2020 state flow is shown in Figure 2. 3,227 publications were identified and screened for duplicates. This was followed by title and abstract screening. Duplicates were found by applying a self-written Matlab script. Duplicates found later were added to the number of “duplicate records removed” accordingly. After screening the abstracts, 62 publications remained. These full texts were checked against the exclusion criteria. A snowball search based on the cited authors and an Internet search of the lead authors in each publication yielded 16 additional publications of interest. A total of 39 articles and 6 reviews were included in this work.

### 3.1 Excluded publications

Due to the rapid progress in research topics related to robotics and eye tracking, this section provides a brief overview. Especially telepresence robots, social robots, and the use of hBCI and BCI were found to be of growing research interest. Rehabilitation and surgical assistance were mentioned as possible applications. The 36 excluded publications were used to identify these topics.

Six publications were excluded due to their publication type. Three of these were dissertations. Alsharif’s Ph.D. thesis can be partially represented in her publication, which is referred to later in the included articles (Alsharif, 2017). Shahzad and Mehmood (2010) presented a master’s thesis on controlling articulated robot arms using eye tracking. (Nunez-Varela, 2012), focusing on task-driven object manipulation by gaze locations on the object (intention read from gaze). The MyEccPupil (HomeBrace GmbH) is a commercially available eye tracking controller for a wheelchair-mounted robotic arm. Although other eye tracking based systems were searched worldwide, no other systems could be found. Two related patents were found (Payton et al., 2013; Norales et al., 2016).

Telepresence robots (Mejia and Kajikawa, 2017; Zhang et al., 2019), smart or electric wheelchairs (Cowan et al., 2012; McMurrough et al., 2012; McMurrough et al., 2013; Jiang et al., 2016; Meena et al., 2016; 2017; Callejas-Cuervo et al., 2020; Edughele et al., 2022), social or humanoid robots (Ma et al., 2015; Mejia and Kajikawa, 2017; Nocentini et al., 2019; Gonzalez-Aguirre et al., 2021; Kyrarini et al., 2021; Liang and Nejat, 2022; Robinson et al., 2022) are controlled by gaze. Such programs greatly facilitate the daily life of people with upper limb impairments. The movement of other devices such as cameras by gaze can help in the control of surgical robots for laparoscopy (Zhu et al., 2010; Despinoy et al., 2013). Similar to direction corrections as in surgical context gaze can be used to interpret human intentions. Human intend detection were used to interpret the desired action the robot should accomplish to enhance the performance of a shared control (Jain and Argall, 2019). A human reflex also includes the dilatation of the pupil in certain events and can be used for shared control improvement (Aronson et al., 2018; Aronson and Admoni, 2019; 2020; Aronson et al., 2021).

Eye tracking is a versatile technology and is used, among other things, to detect whether the participant’s gaze is fixated on the monitor presenting the robot control interface Postelnicu et al. (2019). Projects such as ASPICE focus on a selection of input modalities to match the needs of the participants in order to assist patients with different tasks in the form of a hBCI (Cincotti et al., 2008). A robotic dog (AIBO) was used to test the modalities.

A variety of robotic applications can be found in rehabilitation. In cases of stroke rehabilitation, the robots are used to move the extremities through gaze implication, which is detected by EEG, EOG and VOG (Kirchner et al., 2013; Maimon-Dror et al., 2017; Crea et al., 2018; Shafti et al., 2019; Shafti and Faisal, 2021). Currently, it has been shown to restore hand function, but the “fluent, reliable and safe operation of a semi-autonomous whole-arm exoskeleton restoring ADLs” have to be demonstrated (Crea et al., 2018). Approaches for home physical therapy can be realized by exoskeletons (Kirchner et al., 2013; Pedrocchi et al., 2013; Maimon-Dror et al., 2017) or robotic gloves (Noronha et al., 2017; Shafti et al., 2019; Shafti and Faisal, 2021). Assisting with drinking tasks using an exoskeleton is mentioned in Crea et al. (2018). Other tasks include food preparation using virtual reality and an exoskeleton (Novak and Riener, 2013) or drawing on a screen without a robotic system (Shehu et al., 2021).

### 3.2 Related reviews

This section provides a brief overview of reviews in related research areas. A review of input modalities has been conducted by Clark and Ahmad (2021), who present eye tracking, computer vision and EEG approaches, emotion recognition, gestures, and lie detection. Advantages and challenges are presented along with an extensive literature review. It is aslo stated that non-verbal and non-touch based approaches are important for the future development of intuitive and natural feeling robot control. Esposito et al. (2021) reviewed biosignal-based Human Machine Interaction (HMI). These were biopotential biosignals such as EEG, muscle-mechanical motion, body motion, and hybrid approaches. This review focused on the use of EOG, among others. Ramkumar et al. (2018) presented an overview of the review classification of EOG-based Human Computer Interfaces (HCI) considering data from 2015 to 2020. In addition, Schäfer and Gebhard (2019) compared five hands-free input modalities that are important in robotic arm control research. Dünser et al. (2015) presented a similar approach and compared four input modalities. The results of both works are discussed in Section 4.3.1. In addition, a comparison of BCI and eye tracking in eye typing studies using a spelling program with people with severe motor impairments (Pasqualotto et al., 2015).

Robotics in healthcare is a promising approach addressing the shortage of skilled workers. The goal is to support patients in physical therapy and everyday life as well as caregivers in their daily tasks. Human recognition, emotion and speech recognition are used for the realization. Kyrarini et al. (2021) present robots in different scenarios, such as care robots like Pepper, hospital robots that help with logistics, physical therapy robots like exoskeletons and walking aids and finally assistive robots like FRIEND, Jaco 2, and Baxter.

Finally, a review of wearable interaction was found that defined head and eye movements used to control wearables (Siean and Vatavu, 2021). The results showed that there is limited research on the accessibility of such systems for people with motor impairments. The main findings of this review revealed four suggestions for future research. 1. exploring a variety of wearables, as the current focus is on head wearables, 2. multimodal input modalities and input modalities that maximize motor abilities, 3. more user studies, and 4. IoT.

### 3.3 Included works

The literature found is the basis for answering research question RQ1: “What approaches have been explored in the field of gaze-controlled robotic arms to assist people with (severe) upper limb impairments?”. Table 4 lists all articles that met the inclusion criteria. It is divided into three sections called telemanipulation, directional gaze, and object-oriented gaze. This separation was chosen because the interaction between the participant and the system can vary. Telemanipulation describes the use of a display that allows visual feedback and the presentation of buttons to manipulate the robot. Directional gaze approaches are based on looking in a direction to move the robot. Switching between robot manipulation dimensions (e.g., *x*-*y* axis to *x*-*z* axis) or grasping mode is achieved by including blinking or using additional input modalities. Object-oriented gaze entails the need to either determine gaze in 3D-space, implement object detection algorithms, and adapt trajectory planning to automate the task. For all three categories, we distinguish between 1) assistive systems in everyday life and 2) miscellaneous applications, which include industrial applications, comparative work, and tasks that are not performed in everyday life.

**FIGURE 3:**
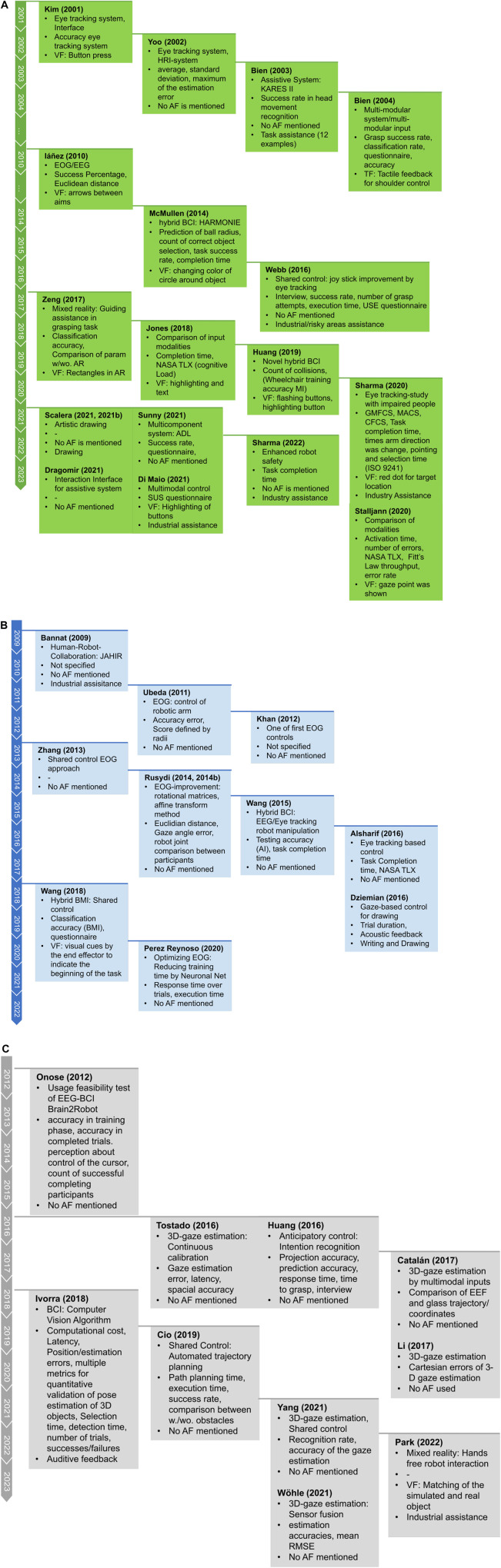
Presented characteristics in all subfigures: Research focus, evaluation parameters/metrics, use of artificial feedback, optional: use in non-assistive living environment. **(A)**Publications with focus on telemanipulation. **(B)**Publications with focus on directional gaze. **(C)**Publications with focus on object oriented gaze.

In Table 4, the Technology column is divided into the type of eye tracking device used, the location of the device, and the type of robotic arm mentioned in each publication. Details to the composition of the system in the online table (Fischer-Janzen, 2023). As can be seen, most systems use either eye tracking glasses (head-worn, 17 of 39 publications) or remote eye tracking devices (12 of 39 publications). Approaches using EOG devices are represented by 9 out of 39 publications. Robotic arms are divided into industrial robots (R: 11/39 pub.), collaborative robots (CR: 7/39 pub.), assistive robotic arms (ARA: 9/39 pub.), educational robots (EDR: 4/39 pub.), and modular prostheses (PR: 2/39 pub.). As a second characteristic, robot motion was divided into pointing to objects or positions (12/39 pub.), grasping objects (10/39 pub.), and performing tasks (17/39 pub.). Most authors chose one task, such as ADL, to proof the functionality of their system. If the system did not have an effector to grasp the object or to interact with it in some other way (e.g., with a magnet), it was reported as “point.” If the object was grasped but no further interaction was reported, the classification “grasp” was chosen. Note that “task” also describes pick and place tasks, as many tasks can be accomplished by this object manipulation, such as setting a table. The type of eye movement was evaluated to determine the effects of the Midas Touch Problem or similar effects. In total, 32 of the 39 publications used fixation based approaches (F). 4 of 39 publications also used saccades. In addition, blinking (B) was detected in seven publications. The workspace in which the robot is to be controlled was divided into 2D, moving in a plane and 3D, which is necessary, for example, to place objects in shelves. Approaches of recognition 2D gaze in a 3-dimensional control of a robot is challenging. Most of the authors presented solutions to control the robot in 3-dimensional Cartesian space (29/39 pub.). The last three characteristics describe the characteristics of the conducted user studies (28/39 pub.). Six studies included handicapped participants. In addition, Figure 3 presents a timeline of all included works and provides insights into the research focus, measurements, and application of artificial feedback.

#### 3.3.1 Telemanipulation

Telemanipulation is usually realized by displaying digital buttons on a screen. They represent either directions and rotations of the Tool Center Point (TCP) or the robot gripper, individual joint positions, or complete tasks. In the latter case, the robot will act independently to achieve the goal of the task. In terms of the items shown in Table 4, most telemanipulation motions are described as “task” because complex tasks can be accomplished by manually controlling the robot using directional buttons.

The tasks used to test these telemanipulation systems were drinking (Huang et al., 2019; Stalljann et al., 2020), opening doors (Dragomir et al., 2021), drawing (Scalera et al., 2021a; b), printing cloths (Sharma et al., 2020; 2022), assisting with industrial tasks in shared workspaces (Di Maio et al., 2021), serving a meal or drink, picking up objects, changing CDs or tapes, making tea, and shaving (Bien et al., 2004).

The development of eye tracking based robot control began in early 2000 (see Figure 3; Kim et al. (2001)) presented the first system based on a self-developed eye tracking system and interface to control an industrial robotic arm using on-screen buttons. Similar systems have been presented to control a robotic arm via a GUI (Yoo et al., 2002; Sunny et al., 2021). Using a GUI means that the person does not need to have the same field of view as the robot because the scene can be displayed on the screen. This is helpful for people that are bedridden. For example, an EEG/EOG-based system capable of controlling a robot from another building was presented by Iáñez et al. (2010).

Although the display used in such telemanipulation approaches provides a solid basis for using either head-mounted or remote eye trackers many approaches have been found that use or comparing multimodal inputs. KARES II was one of the first systems using multiple input modalities such as eye-mouse, haptic suit, face recognition and EEG Bien et al., 2003, Bien et al., 2004. For this purpose a PUMA-type robotic arm, an eye tracking setup, and a haptic suit were developed. In this work the mouth was tracked to estimate drinking intent, requiring the combination of several input modalities. The HARMONIE system demonstrates an approach using intercranial electroencephalography (iEEG) signals and eye tracking as a hBCI (McMullen et al., 2014). Eye tracking was used to move a cursor and select targets.

In the experiment by Jones et al. (2018), the robot was used to play chess at different levels of difficulty. The two input modalities, eye tracking and joystick, were compared. Webb et al. (2016) presented a system that enhanced teleoperation for a robot by using gaze to manipulate it toward a fixated object. The object was approached using a joystick controller. A comparison between head and gaze performance in a robot control task evaluated different applications between continuous and discrete control events (Stalljann et al., 2020). This study demonstrated the importance of including people with disabilities, as there were measurable differences between the quadriplegic and able-bodied participants.

In several approaches, the use of multiple subsystems was targeted. In particular, the control of an electric wheelchair and an ARA, as done by Dragomir et al. (2021) and Huang et al. (2019), is of interest because it provides more mobility to the user. The design philosophies of such systems should include task-oriented design, “human friendliness” including safety precautions, and “modularization of subsystems” as stated by Bien et al. (2004). They combined a robotic arm, an electric wheelchair, and a mobile platform.

Another important advantage of the display is its ease of use for displaying visual feedback. In the approach of Zeng et al. (2017), the hBCI was enhanced by using Augmented Reality (AR) inputs to correct an eye tracking pick-and-place task. In this AR environment, colored rectangles were presented as visual feedback to help sort colored objects. Others were visualizations of virtual button presses (Kim et al., 2001), tactile feedback in a multimodal shoulder control (Bien et al., 2004), and highlighting as well as text descriptions (Iáñez et al., 2010; McMullen et al., 2014; Jones et al., 2018), as shown in Figure 3.

#### 3.3.2 Directional gaze

Directional gaze is defined as rapid eye movements (saccades) with additional optional fixation events in a particular direction. Directional gaze can be helpful in improving the performance of the system, as quite large areas can be discriminated for a given movement. For example, Alsharif et al. (2016) demonstrated one of the first systems that worked without a screen and with gaze gestures, in which participants could control a robotic arm with a specific cue of eye movements. The directional control allowed the user to perform various pick-and-place tasks. The end-effector could be translated and rotated through the interface.

Most of the grouped systems performed pick-and-place tasks (Khan et al., 2012), but this straightforward approach has also been tested with other tasks, such as writing and drawing (Ubeda et al., 2011; Dziemian et al., 2016), according to the principle “look to the right, draw a line to the right.” Other approaches let the robot perform a free trajectory in which the user visually focused on visual markers on a wall and the robot followed by interpreting EOG signals (Zhang et al., 2013). Five other publications on EOG are mentioned in this category (Khan et al., 2012; Ubeda et al., 2011; Perez Reynoso et al., 2020; Rusydi et al., 2014a; b). One challenge is matching the 3D motion of the robot to the 2D motion of the eye. A solution was presented by (Perez Reynoso et al., 2020) by adapting the system to user-specific parameters using a fuzzy inference system. The response time was reduced and 3-dimensional movements could be performed with 2-dimensional eye inputs by using fuzzy classifiers. With this advanced technology, the system is able to use specific coordinates to separate the signal into multiple locations in a workspace.

Other multimodal approaches, such as Wang et al. (2015), combined an EEG system with an eye tracking device by implementing a trained HMM to improve the performance of the hBCI. The authors stated: “Our goal was to enable flexible and unscripted control while ensuring high reliability.” A multimodal system including gaze and EEG was used to perform a multiple obstacles grasping task. Gaze was used to correct the robot’s trajectory and EEG to control the speed of the end effector Wang et al. (2018). Especially in shared workspaces, adding speech and buttons as input can improve accessibility in assembly tasks (Bannat et al., 2009; Rusydi et al., 2014).

#### 3.3.3 Object-oriented gaze

Object-oriented gaze describes the fixation on an object to indicate an interaction to the system. This can improve the user experience because humans tend to look at the object we want to interact with. In addition, the user is not forced to look away since most of the task execution is automated and the robot is not controlled by looking in a certain direction as in the previous section. This leads to the need for computer vision to extract features from the objects as it has been applied in most cases.

Tasks such as picking ingredients with intention recognition (Huang and Mutlu, 2016), interacting with everyday objects (Onose et al., 2012; Ivorra et al., 2018), or grasping a pair of scissors (Yang et al., 2021) have been realized using eye tracking techniques. The AIDE project uses EEG and EOG to move a robotic arm. The state-of-the-art algorithm uses AI to improve object selection from textureless objects in real time. Mouth poses have been used to improve user safety. The solution can be adapted to control an electric wheelchair (Ivorra et al., 2018).

Object-oriented gaze is a robust solution to the problem of grasping objects in 3D space. The user does not have to switch modes to move between x-y and *x*-*z* axes. 3D-gaze estimation can be used to identify the location of objects (Tostado et al., 2016; Li et al., 2017; Wöhle and Gebhard, 2021). This information facilitates robot trajectory planning. An algorithm for continuous calibration of the eye tracking device to track the gaze as a 3D point in a scene was presented by Tostado et al. (2016) and realized with a stereo vision camera and machine learning approaches. This system is useable for people with “strabism or other eye alignment defects.” Magnetic, Angular Rate, and Gravity (MARG) sensors and eye tracking were fused to estimate head position in the work of Wöhle and Gebhard (2021). Yang et al. (2021) introduced a system using Apriltags to facilitate joint control with the robot. Points of interest were detected by gaze. Intention detection was used to determine whether an object should be grasped at a particular location. For 3D gaze estimation, head movements were additionally tracked. By combining artificial stereo vision and eye tracking as an input device Cio et al. (2019) showed that the system performed well in a human-guided grasping task. Even in the presence of obstacles, a task success rate of 91% was achieved. Catalán et al. (2017) presented a multimodal control architecture that uses two eye tracking devices to estimate the location of objects in a scene.

In contrast to telemanipulation using a screen, an approach to the use of mixed realities has been found that also allows the use of artificial visual feedback (Park et al., 2022). This system has been used in industrial applications to realize a shared workspace. Through gaze selection, objects can be defined to be removed by a robot from a given space. In a service application, a system for predicting user intent in a drink mixing task was developed.

## 4 Discussion

This section discusses research questions RQ2 and RQ3. To answer these questions, a more general overview of the behavioral and technical interpretation of eye movements is presented. A comparison of input modalities, future trends, and open questions will answer RQ3 to increase transparency in this diverse field.

### 4.1 Interpreting gaze in robot control

As shown, most researchers use fixation to define events. In this context, fixation is defined as focusing on an object or direction. The resulting risk of the Midas Touch Problem can be reduced by a well choosen dwell time of about 200–700 ms. The Midas Touch Problem is reported in several included publications (Bien et al., 2004; Drewes and Schmidt, 2007; McMullen et al., 2014; Velichkovsky et al., 2014; Alsharif et al., 2016; Dziemian et al., 2016; Webb et al., 2016; Meena et al., 2017; Jones et al., 2018; Stalljann et al., 2020). In some experiments, dwell time leads either to pleasant or irritating situations. It was interpreted as entertaining in the experiment of Bednarik et al. (2009) as it was seen as a challenge in a game or frustrating since it caused wrong decisions in object manipulation (Jones et al., 2018). Saccades are rarely used. One reason found is that saccades are highly noisy, unintentional, and not goal-directed, which makes them difficult to interpret (Dziemian et al., 2016). Saccadic movements are used by Scalera et al. (2021a) to determine the trajectory of the eye movement and implement virtual brush strokes, which were then interpreted by the robot. Saccadic movements are also used to determine electrical potential changes in EOG signals (Khan et al., 2012).

Some approaches use different input modalities such as gaze gestures or blinks, although these can be highly intuitive and enrich the received gaze information (Duguleana and Mogan, 2010; Iáñez et al., 2010; Alsharif et al., 2016; Dziemian et al., 2016). A gaze gesture is described as a sequence of eye movement elements (Drewes and Schmidt, 2007) or as “the number of strokes performed in a predefined sequence” (Alsharif et al., 2016) and includes fixations and saccades as well as blinks and winking. If these sequences are easy to remember it may have an impact on usability.

In addition to the behavioral aspect of interpretation, the provision of interpretable data to the robot must be ensured. Not every publication provided detailed information about the software architecture. The solution was usually divided into two phases: 1) filtering and analysis of the eye-tracking data and, if used additional cameras and sensors and 2) robot trajectory planning. In the first step, OpenCV was used several times to detect the position of the pupil. Other systems like Pupil Core from Pupil Labs and the eye trackers from Tobii already provide an API to receive this data. The trajectory planning was mostly done with ROS MoveIt or GraspIt. The first ROS distribution was released in 2010. Therefore, in the first publications mentioned, ROS did not exist, so it is most likely that the robot was controlled directly by programmable logic controllers (PLC). New Python-based toolkits, modules, and environments are being developed to facilitate the control of robots and the implementation of AI models as it was presented (e.g., PyBullet, OpenVINO, Open AI Gym, and many more).

### 4.2 Benefits, challenges and limitations of eye tracking

Gaze is considered an intuitive control modality because it requires low cognitive load, is proactive, and directly correlates with action intentions (Tostado et al., 2016). It provides natural, effortless, and rich information that can be interpreted by the robot (Li et al., 2017; Zeng et al., 2017), especially since humans tend to look at objects they want to interact with (Li et al., 2017). When designing interfaces for assistive devices, it is important to consider people’s ability to retrain their motor functions, which leads to different approaches solving a challenge. During the training phase, the setup may be perceived as unnatural and distracting, resulting in additional cognitive load (Tostado et al., 2016). This may change over time. Considering that the system will be worn for several hours in real-world applications, such as head-mounted eye tracking devices, the system should be lightweight and adjustable to reduce discomfort and pressure on the head (Wöhle and Gebhard, 2021). In terms of social factors and the individuality of each user, there are important influences that challenge head-mounted and remote eye trackers. Parts of the environment are usually included in the scene video recorded by the eye tracking camera, which shows limitations regarding privacy regulations (Wöhle and Gebhard, 2021). As stated by the European Data Protection Board, the increased use of smart cameras today leads to a large generation of additional data to the captured video itself. This leads to an increased risk of secondary use for unexpected purposes such as marketing and performance monitoring (Jelinek, 2019). Especially in combination with the use of the robot in ADL, such as for maintaining personal hygiene, ways must be provided to avoid the monitoring of such sensitive data. According to a 2020 report, more than half of humanity has either myopia or presbyopia (WHO, 2019), requiring corrective devices such as contact lenses or glasses. Most eye tracking devices are not useable with glasses or lead to higher inaccuracies (Schäfer and Gebhard, 2019; Edughele et al., 2022).

Inadequate accuracy is often reported for eye tracking devices (Huang and Mutlu, 2016; Jones et al., 2018; Stalljann et al., 2020). The signal-to-noise ratio (SNR) in EOG and VOG is sometimes insufficient (Rusydi et al., 2014; Dziemian et al., 2016; Wang et al., 2018; Huang et al., 2019; Schäfer and Gebhard, 2019). This makes it difficult for users to complete the task and limits bandwidth, resulting in incorrect button and object selection (Dziemian et al., 2016; Huang and Mutlu, 2016; Wang et al., 2018). In VOG and IOG applications, head movements can lead to moving areas of interest (Edughele et al., 2022) due to limited performance of gaze-mapping algorithms (Yang et al., 2021). In experiments, this results in a limitation of head motion (Yang et al., 2021). Other limitations include lighting conditions, camera field of view, and object overlap (Esposito et al., 2021). Eye tracking glasses should be calibrated regularly or secured with a strap, as slippage can lead to errors (Stalljann et al., 2020). A common problem with IMUs or gyroscopes built into the glasses is DC offset. In gyroscopes, magnetic materials can also cause data drift. They are often used to track head or body movements (Schäfer and Gebhard, 2019; Wöhle and Gebhard, 2021). EOG signals must be filtered and segmented to obtain the desired eye movements for control, as unintended eye movements are also recorded (Huang et al., 2019). Gaze directions are difficult to track in previous work (Huang et al., 2019), but solutions are being found in current work (Perez Reynoso et al., 2020). Information is lost through filtering (Iáñez et al., 2010). One reason why EOG signals are easy to use is the linear relationship between signal and eye movement displacement (Rusydi et al., 2014).

#### 4.2.1 Comparison of input modalities

**FIGURE 4:**
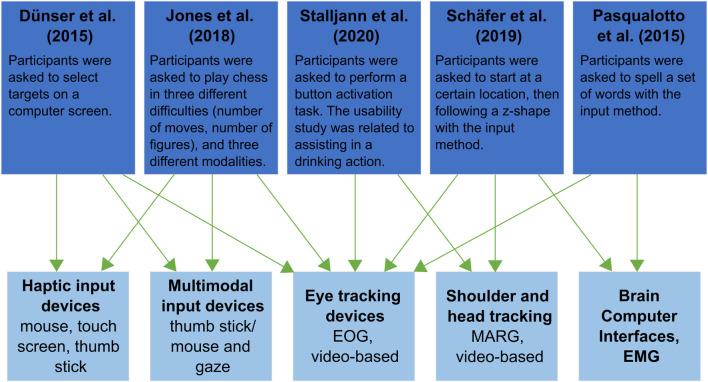
Identified comparative studies describing the task and the used input modalities.

The decision of which input modality is more appropriate depends strongly on the technology used, the goal of the task, and the control algorithm. A number of comparative studies with different experimental setups were identified in this review (Dünser et al., 2015; Pasqualotto et al., 2015; Jones et al., 2018; Schäfer and Gebhard, 2019; Stalljann et al., 2020). Figure 4 shows the articles and the input modalities. The task description is given to provide a deeper insight into the study design.

Overall, VOG and IOG were rated more positively than the other modalities. Positive aspects were identified as subjective rating (low workload/cognitive load (Pasqualotto et al., 2015; Stalljann et al., 2020), comfort (Schäfer and Gebhard, 2019), and robust functionality (Schäfer and Gebhard, 2019). Pasqualotto et al. (2015) suggested its use as a communication device, similar to Schäfer and Gebhard (2019), who stated its use for discrete events, such as trigger events, based on the traceability of fast eye movements. Negative aspects have been reported in poor performance with the Fitt’s law test, which is associated with low accuracy (Dünser et al., 2015; Jones et al., 2018). It describes the relationship between the time it takes to move quickly to a target area and the size of that target (Dünser et al., 2015).

Contrasting results have been found with other modalities such as the thumbstick (Jones et al., 2018) and the mouse (Dünser et al., 2015). Jones et al. (2018) found better performance when combining input modalities than when using eye tracking alone, as it was faster and found better results in workload. However, Dünser et al. (2015) reported worse results for the eye tracking approach than for the mouse and touchscreen in both objective and subjective measures. One reason for this may be that most participants have used one of these before (Yoo et al., 2002; Jones et al., 2018). It is difficult to determine whether the differences in parameters are due to learning effect or pure perception.

Head tracking using a Magnetic, Angular Rate, and Gravity (MARG) sensor has also been positively evaluated (Schäfer and Gebhard, 2019; Stalljann et al., 2020). This type of sensor can provide comprehensive data on humans movement. Stalljann et al. (2020) presented that button activation to perform the task was less demanding, strenuous and frustrating with this sensor. Shoulder or head control is considered less intuitive than hand control because the information density is reduced. It is also mentioned that it can be more natural than other input devices (Bien et al., 2003). In comparison, shoulder control was found to be a suitable candidate for moving a robotic arm in continuous control and was rated better than eye tracking in one case (Stalljann et al., 2020). The patient with tetraplegia was able to perform better with eye tracking as measured by the lower error rate. The difference to the MARG sensor was not significant in the group of able-bodied participants.

EEG, EOG, and BCI were rated lower than all other input modalities due to low signal transmission (Schäfer and Gebhard, 2019), additional hardware required (Schäfer and Gebhard, 2019), and more effort and time required to perform the task (Pasqualotto et al., 2015).

### 4.3 Future trends

The result of research question RQ3 showed an increased mention of the following topics: multimodal control, including 3D gaze estimation and hBCI, and shared control. The authors see high potential in these topics as they can reduce the error rate of eye tracking devices used as stand-alone systems, improve the usability of such systems, and address individual needs.

#### 4.3.1 Multimodal control inputs

Multimodal control inputs can improve the user experience for each participant. They are combined to adapt to the degree and type of impairment (Bien et al., 2004; Siean and Vatavu, 2021) and to the task (Bannat et al., 2009; Di Maio et al., 2021), increasing the intuitive control of the complex system (Bannat et al., 2009; Li et al., 2017; Sharma et al., 2022). The system of Bien et al. (2004) presents a hierarchy and design for different levels of disability. The user was able to move the system with the shoulder if the upper body motor functions were still intact. If the patient has a progressive disease, he can later switch to eye tracking control. The use of multiple input modalities leads to improved performance (Esposito et al., 2021), such as combining gaze and joystick control (Webb et al., 2016) or gaze and head tracking (Kim et al., 2001).

In this work, 6 of the 39 identified and included papers involved the use of hBCI that included eye tracking devices. Eye tracking was used for rapid intention detection, such as moving the end-effector in a particular direction (Wang et al., 2015; 2018), for object pose estimation (Onose et al., 2012; Ivorra et al., 2018), or for triggering switch events (Zeng et al., 2017). For detailed information, we refer to Esposito et al. (2021); Hong and Khan (2017); Nicolas-Alonso and Gomez-Gil (2012); Pasqualotto et al. (2015); Trambaiolli and Falk (2018).

3D gaze estimation can be achieved by combining different input modalities. By estimating the intersection of the lines of sight, we can determine the 3D gaze position with respect to the scene camera in head-mounted glasses (Tostado et al., 2016; Yang et al., 2021). Due to head movements, the point on a world coordinate system needed for robot manipulation is not stable. Two approaches that combine head tracking with eye tracking have been identified. The first approach used motion detection from video cameras placed around the user (Onose et al., 2012; Wöhle and Gebhard, 2021; Yang et al., 2021). The second approach uses accelerometers, IMUs and MARG sensors placed on the user’s neck and head (Wöhle and Gebhard, 2021). The latter is mostly used as a control based on head motion unrelated to 3D gaze estimation (Schäfer and Gebhard, 2019; Stalljann et al., 2020).

#### 4.3.2 Shared control

A synonym for shared control was found to be described as semi-autonomous robot control. The robot can act and perform movements or tasks automatically, but is dependent on a user. The goal is to realize a comfortable and intuitive control of the device (Bannat et al., 2009; Li et al., 2017; Sharma et al., 2022). One solution is to incorporate intention recognition into the design process (Bien et al., 2004; Huang and Mutlu, 2016; Shafti and Faisal, 2021). Intention can be read by Areas of Interest (AOI) in eye tracking approaches, such as the handle of a cup when the user intends to grasp it or by tracking facial features. Further literature on this area of research can be found in Jain and Argall (2019); Aronson and Admoni (2019); Bonci et al. (2021), and Hentout et al. (2019).

Task-oriented design is one of the key benefits of shared control. Activities of daily living are composed of multiple subtasks. For example, drinking can be divided into reaching for and grasping a bottle, filling the glass, placing the bottle, taking the glass and reaching toward the user. A human will decide at which step to stop, and it is not necessary to perform them all in one go. In most of the presented approaches, the task is realized by the user fixating the object and correcting the system (Bien et al., 2003; 2004; McMullen et al., 2014; Wang et al., 2015; Huang and Mutlu, 2016). A comparison of the resulting degree of autonomy is shown for a feeding task by Bhattacharjee et al. (2020). The main finding is that the user should always feel in control of the situation and control.

One way to implement shared control is to use of AI, as has been done in several works. Detecting of an object can be easily done using models such as YOLO or RetinaNet (Ivorra et al., 2018; Park et al., 2022). Neural networks have already been implemented to learn robot motion (Bien et al., 2004), to model EOG functions (Perez Reynoso et al., 2020), to model this eye-hand coordination behavior during grasping (Li et al., 2017), or for face recognition (Sharma et al., 2022). The use of AI helps to automate tasks. Especially in robotics, but also in behavioral science, these models are becoming increasingly important.

### 4.4 Open questions

To conclude RQ3, open questions were uncovered that will be necessary progressing in the design process of the eye tracking control system for robotic arms. Frequently mentioned research topics focused on performance measurement, inclusion of people with disabilities in studies, and the use of artificial feedback to improve the usability and accessibility of the system.

#### 4.4.1 Assistive robotics performance measurements

How the performance of an ARA is measured is highly dependent on the system, the domain of origin (e.g., computer science), and the study design. It was found that the most common parameters reported in the included papers were task completion time and success rate (Anke Fischer-Janzen, Overview of Measurements, URL: https://github.com/AnkeLinus/EyeTrackingInRobotControlTasks/blob/main/OverviewOfMeasurements.md, last accessed: 09.02.2024). These parameters are not comparable between studies because of differences in design. All reported parameters were divided into task-related parameters, computational parameters and empirical parameters. Computational parameters are mostly measured as accuracy (Bien et al., 2004; Onose et al., 2012; Wang et al., 2015; Li et al., 2017; Zeng et al., 2017; Huang et al., 2019; Yang et al., 2021). In the case of object recognition, parameters such as prediction accuracy, projection accuracy (McMullen et al., 2014; Huang and Mutlu, 2016; Ivorra et al., 2018), recognition rate (Zhang et al., 2013; Yang et al., 2021), and reaction time (Huang and Mutlu, 2016) are used. Computational cost, as reported by Ivorra et al. (2018), was used as a way to rank different algorithms. Task-related parameters are most often given as task completion time and success rate. Counting different events also helps the reader to estimate the challenges in the system. For example, authors mention the number of required gestures, which indicates the effort required by the user (Alsharif et al., 2016), or the number of collisions when there are obstacles (Huang et al., 2019). In the case of eye tracking, gaze parameters such as dwell time (fixation duration), gaze estimation accuracy, or areas of interest are controlled (Holmqvist and Andersson, 2017; Edughele et al., 2022). Other errors have been reported by authors such as Euclidean and Cartesian errors to describe the drift from the intended position to the robot position (Tostado et al., 2016; Li et al., 2017; Perez Reynoso et al., 2020; Wöhle and Gebhard, 2021). We want to show how to ensure better comparability between studies by specifying exemplary parameters. The completion time itself depends on several parameters. Dividing the task into several steps has been found to be helpful, such as different empirical and computational parameters, (e.g., reaction time of the user and the computation and the execution time with the robot). Reproducible results can be obtained by additionally specifying the trajectory length and the maximum speed of the robot. Boundary conditions for reproducibility were missing in some publications.

User satisfaction is evaluated through subjective measurements. The following tests and questionnaires have been identified: NASA TLX (NASA Task Load Index), USE questionnaire (Usability, Satisfaction and Ease of use), SUS questionnaire (System Usability Scale), and QUEST 2.0 (Quebec User Evaluation of Satisfaction with assistive Technology). Non-standardized tests were often used to analyze specific features of the system. This section has been expanded to include the QUEAD (Questionnaire for the Evaluation of Physical Assistive Devices), based on the TAM (Technology Acceptance Model), to provide another test method applicable to assistive robotics. Table 5 should provide a quick overview for new researchers. The question to be answered in future work is to what extent these questionnaires can be used to evaluate performance in an eye tracking based robot control. Although most of the questionnaires are well established in science and hurdles in execution and evaluation have been uncovered, the novelty and therefore the application to this topic needs to be explored.

**TABLE 5:** Standardized questionnaires for subjective evaluation of assistive robotics.

Questionnaire	Applicable for the evaluation of	Number of items	Used in included works	Limitations
NASA TLX Hart and Staveland (1988)	Task-dependent human-centered evaluation of workload	6 items, 100 or 50 point Likert scale	Alsharif et al. (2016); Stalljann et al. (2020)	Failed test of construct validation and varying interpretation between participants (McKendrick and Cherry, 2018)
USE Lund (2001)	Measurement of the subjective usability of a product or a service Gao et al. (2018)	4 categories with a total of 30 items, 7 point Likert rating scale	Li et al. (2017); Webb et al. (2016)	Refinement due to validation enhancement was presented by Gao et al. (2018)
SUS Brooke (1996)	Global assessment of system usability	10 items, 5- or 7-point Likert scale	Di Maio et al. (2021)	Usability cannot be measured in an absolute way (Brooke, 1996). For a detailed insight in the subjective dimensions efficiency, effectiveness and satisfaction other questionnaires should be conducted
QUEST 2.0 Demers et al. (1996)	Satisfaction of an assistive device or service	12 items in two dimensions (Device and Service), 5-point satisfaction rating scale	Yang et al. (2021)	Generic Assessment, items related to certain Assistive devices may be absent, yet can be added. Demers et al. (2002)
QUEAD Schmidtler et al. (2017)	“Subjective perception of usability and acceptance of a new physical assistance system or control mode.” - Schmidtler et al. (2017)	5 categories with a total of 26 items, some categories can be used separately. 7- or 5-point Likert scale. Tested on an assistive robot	—	Till now no QUEAD scale benchmark
Non standardized questionnaires	Varying content depending of the researchers interest	—	Sunny et al. (2021); Huang and Mutlu (2016); Onose et al. (2012); Jones et al. (2018); Stalljann et al. (2020)	Biasing the participants should be avoided. Significance, etc., can vary

#### 4.4.2 User-centered design

In this review, six of the 39 publications included people with physically disabilities. A lack of inclusion of disabled people in such studies can be seen in other areas, such as the accessibility of wearables (Siean and Vatavu, 2021). Participants’ opinions about the system are crucial for improving such systems. Differences in the evaluation between able-bodied and disabled participants have been reported several times (Bien et al., 2004; Cincotti et al., 2008; Pasqualotto et al., 2015; Stalljann et al., 2020). For example, Stalljann et al. (2020) found that healthy participants could easily switch between controls and interpret the artificial feedback well. This was much more difficult for the tetraplegic participant. Onose et al. (2012) cited difficulties caused by changing postures in a multi-day study. In particular, severely immobile users may find it difficult to remain in the field of view of stationary eye tracking devices (McMullen et al., 2014).

According to Lulé et al. (2008), the decision against life-prolonging treatments in ALS patients is based on the fear of loss of autonomy caused by lack of mobility and aggravated communication. This has a major impact on the Quality of Life (QoL). Eye tracking based controls can help to increase the possibilities of an autonomous life (Edughele et al., 2022). Moreover, everyday use of such systems poses risks and challenges. Regarding an ARA mounted on an electric wheelchair, the needed instruments of a tetraplegic person can include respiratory and gastric catheters as well as communication devices (Edughele et al., 2022). Therefore, the movement of the robot has to be done carefully in close proximity to the person so as not to interfere with the tubes and cables. In addition to medical challenges, the research in this area should be guided on what activities and needs should be addressed and are necessary to provide an improvement in QoL for each individual. The performance of ADL with the use of ARA may be a promising solution to improve QoL. Care is mostly provided by family or professional caregivers. They take care of personal hygiene, dressing, medication, and in the case of assistive devices such as the ARA, maintenance and daily setup of the system (Chung et al., 2013). Especially when the patient has invasive devices, a clean environment is necessary. The system should be easy to keep sterile. In addition, the system should be easy to deploy to further reduce time pressures on caregivers.

Based on these impressions, the question arises as to what other effects have not yet been found in the case studies. Further studies need to be conducted with real-world robots controlled by eye tracking to assess the effects on impaired and able-bodied people. In these systems, multiple users are involved in creating a user-friendly design. The design steps should include additional input from caregivers and family members to ensure ease of use. Due to the novelty of the technology no details could be found.

#### 4.4.3 Artificial feedback

Artificial feedback is well studied in the field of electric prostheses or other computer-based user interfaces. It helps to provide an easy-to-interpret signal for closed-loop interaction between the user and the robot, and provides a good user experience when applied correctly. The question arises as to how such feedback can be used in robotic applications and eye tracking. Figure 3 shows the use of artificial feedback in the form of auditory, visual, and tactile feedback in the included publications. Summarizing all included publications, 25 authors reported no use of feedback. 11 used visual feedback, two used auditory feedback and in one publication was tactile feedback used.

In the telemanipulation systems group, half of the authors used visual feedback to indicate system performance to the user. In most APIs, button click visualization, colored boxes, outlines, and bounding boxes are easy to program and provide visual feedback to the user about their interaction with the system. Transferring these feedback methods to a controller without a display seems complicated and may be unnecessary if the robot responds quickly. This may be one reason why most studies have not suggested the need for further artificial feedback. However, it can provide important information to the user, resulting in higher user satisfaction (Zeng et al., 2017). In the identified publications, two authors used visual feedback without the use of a display. Wang et al. (2018) used the robotic arm itself to provide visual cues by moving the end-effector to trigger each task. Park et al. (2022) used virtual objects displayed in augmented reality to visualize the movement of the object, which was then performed by the robot. The use of virtual and augmented reality allows this discipline to easily integrate visual ways to easily integrate visual feedback into real-world tasks.

Auditive feedback can be easily applied when using a tablet (Edughele et al., 2022). Examples of the use of auditory feedback were provided by Ivorra et al. (2018) and Dziemian et al. (2016). In these papers, a brief tone indicated either the selection of an object (Ivorra et al., 2018) or the successful selection of a movement command (Dziemian et al., 2016). Design rules for auditory feedback can be found in the work of Tajadura-Jiménez et al. (2018). 

Tactile feedback can be used. With small vibration motors like those used in smartphones. Vibrotactile feedback can be realized, which could be mounted on the eye tracking glasses (Rantala et al., 2014; Fischer et al., 2023). Bien et al. (2004) used it to provide status information to the user. There are several design rules for vibrotactile feedback that should be considered (Gemperle et al., 2001; Choi and Kuchenbecker, 2013; Rantala et al., 2020). These are mostly related to either body-worn devices or integrated into eye tracking glasses.

While this is a good start, more research needs to be done on what feedback is practical and whether it affects user or system behavior.

### 4.5 Limitations

Interest in assistive devices will grow in the coming years due to the needs of the elderly and disabled, as well as the decline in caregivers. Each of the research interests presented above is a vast domain in itself. Therefore, a bias based on the limitation of robotic application cannot be excluded. To reduce this bias and to enrich the discussion, the excluded works were partially included. The focus was on research that considered eye tracking in combination with a robotic arm. Due to the interdisciplinary nature of the field, different results for advantages and disadvantages may be found in literature focusing on only one of the topics.

**FIGURE 5:**
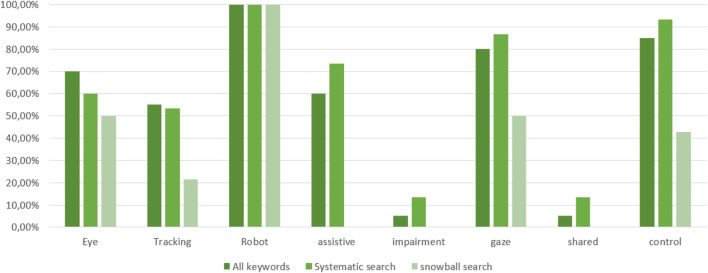
Search term analysis.

The bias was estimated by comparing the keywords given by the authors and the search terms. Figure 5 shows the results for the keywords listed in Section 2. The top 10 keywords used in all publications are 1. robot, 2. human, 3. interface, 4. control, 5. gaze, 6. robotic, 7. eye, 8. interaction, 9. assistive, 10. computer. The reason why 17 relevant publications were found in the snowball search is based on the absence of keywords such as “assistive,” “impairment” and “shared” and the fact that the database was not included in the initial search. Regarding the search terms used in the methods, “tracking” was not among the top 10 keywords in the list of systematically searched papers. The keywords “brain,” “machine,” “BCI” and “EOG” were identified in the snowball search. Since the focus of this paper is on eye tracking based systems, the identification and screening process was not repeated. Regarding the top 10 list of all publications, a new search would not have yielded any new results because most of the terms are synonyms of a previous used search term or would have missed key terms.

## 5 Conclusion

We presented an overview of 39 works with real robotic applications controlled by eye tracking. These approaches require interdisciplinary knowledge from the fields of robotics, human-robot interaction, and behavioral science. The interdisciplinary nature of the field often leads to different keywords for the same topic, making it difficult to find literature. It has been shown that the interpretation of gaze depends on the hardware used, the task the robot has to perform, and the and the stage of the user’s disability. Research interests were found in topics such as multimodal inputs and shared control. Insight was provided on performance measurements, inclusion of disabled people in study design and artificial feedback. Benefits and challenges in terms of eye movement detection methods and various important user parameters were decribed. The comparison of input modalities showed that the optimal devices depend strongly on the user’s abilities. The table presented online (Anke Fischer-Janzen, Study Descriptions, URL: https://github.com/AnkeLinus/EyeTrackingInRobotControlTasks/blob/main/StudyDesciption.md, last accessed: 09.02.2024 and Anke Fischer-Janzen, Overview of Measurements, URL: https://github.com/AnkeLinus/EyeTrackingInRobotControlTasks/blob/main/OverviewOfMeasurements.md, last accessed: 09.02.2024) will be expanded to further facilitate the identification of assistive robotic arm controllers.

## Data Availability

The datasets presented in this study can be found in online repositories. The names of the repository/repositories and accession number(s) can be found in the article/Supplementary Material.
